# Complete metastasectomy after high-dose interleukin-2 (HD IL-2) therapy for melanoma

**DOI:** 10.1186/2051-1426-3-S2-P130

**Published:** 2015-11-04

**Authors:** Melinda Chu, Eddy Hsueh, John Richart

**Affiliations:** 1 Saint Louis University, Saint Louis, MO, USA

## Background

Our aim was to describe our experience with surgery after High-dose Interleukin-2 (HD IL-2) therapy in patients who were eligible for complete metastasectomy after treatment.

## Methods

A retrospective chart review was performed on patients with metastatic melanoma seen at Saint Louis University from January 1999-June 2011 who were partial responders to HD IL-2 therapy (720,000 IU/kg per dose intravenously; 14 doses, 2 cycles per course, maximum 2 courses). Survival estimates for patients that were able to undergo complete metastasectomy after HD IL-2 were compared to established expected duration of survival with systemic therapy alone from the MSLT-1 trial.

## Results

Out of 55 evaluable patients with melanoma who underwent HD IL-2, 8 achieved complete response (14.5%) and 6 achieved partial response, 3 of whom had “resectable partial response” and 3 of whom who had “unresectable partial response”. Of the 3 patients with resectable partial response: Patient 1 had bone, lung, and lymph node (LN) metastases prior to treatment; after HD IL-2, only LN metastases remained. Patient 2 had metastases in the gallbladder, lung, and LNs prior to HD IL-2 and gallbladder lesions only after treatment. Patient 3 had several lung nodules prior to treatment which decreased in number and were amenable to complete metastatectomy after HD IL-2. The actual durations of survival was for Patient 1 (39.5 months) and Patient 2 (17.9 months) were (are) longer than the expected duration of survival for Stage IVc patients (6. 3 months). The expected survival of Patient 3 with Stage IVb disease 9.1 months; actual duration of survival was 19.0 months. The actual duration of survival for Patient 3 was (is) 19.0 months; the expected survival for patients with Stage IVb disease, 9.1 months.

## Conclusions

We show that completing metastasectomy after HD IL-2 had favorable results on overall survival in this small series. The overall survival of these patients was much longer than would have been expected by their M stage if they received systemic therapy alone based on data from MSLT-1 trial. Though systemic therapy was not utilized initially to be a “neoadjuvant” therapy in this retrospective review, we believe the combination of surgery and immunotherapy in the management of metastatic melanoma to eradicate residual tumor burden may improve outcomes and warrants further evaluation.
